# Modeling Brain–Heart Crosstalk Information in Patients with Traumatic Brain Injury

**DOI:** 10.1007/s12028-021-01353-7

**Published:** 2021-10-12

**Authors:** Giovanna Maria Dimitri, Erta Beqiri, Michal M. Placek, Marek Czosnyka, Nino Stocchetti, Ari Ercole, Peter Smielewski, Pietro Lió, the CENTER-TBI Collaborators, Audny Anke, Ronny Beer, Bo-Michael Bellander, Erta Beqiri, Andras Buki, Manuel Cabeleira, Marco Carbonara, Arturo Chieregato, Giuseppe Citerio, Hans Clusmann, Endre Czeiter, Marek Czosnyka, Bart Depreitere, Ari Ercole, Shirin Frisvold, Raimund Helbok, Stefan Jankowski, Daniel Kondziella, Lars-Owe Koskinen, Ana Kowark, David K. Menon, Geert Meyfroidt, Kirsten Moeller, David Nelson, Anna Piippo-Karjalainen, Andreea Radoi, Arminas Ragauskas, Rahul Raj, Jonathan Rhodes, Saulius Rocka, Rolf Rossaint, Juan Sahuquillo, Oliver Sakowitz, Peter Smielewski, Nino Stocchetti, Nina Sundström, Riikka Takala, Tomas Tamosuitis, Olli Tenovuo, Andreas Unterberg, Peter Vajkoczy, Alessia Vargiolu, Rimantas Vilcinis, Stefan Wolf, Alexander Younsi, Frederick A. Zeiler

**Affiliations:** 1 Computer Laboratory, University of Cambridge, Cambridge, UK; 2 DIISM, University of Siena, Siena, Italy; 3 Brain Physics Laboratory, Division of Neurosurgery, Department of Clinical Neurosciences, University of Cambridge, Cambridge, UK; 4 Department of Physiology and Transplantation, University of Milan, Milan, Italy; 5 Faculty of Fundamental Problems of Technology, Department of Biomedical Engineering, Wroclaw University of Science and Technology, Wrocław, Poland; 6 Division of Anesthesia, University of Cambridge, Cambridge, UK

**Keywords:** Intracranial pressure, Traumatic brain injury, CENTER-TBI, Raised intracranial pressure, Raised heart rate

## Abstract

**Background:**

Traumatic brain injury (TBI) is an extremely heterogeneous and complex pathology that requires the integration of different physiological measurements for the optimal understanding and clinical management of patients. Information derived from intracranial pressure (ICP) monitoring can be coupled with information obtained from heart rate (HR) monitoring to assess the interplay between brain and heart. The goal of our study is to investigate events of simultaneous increases in HR and ICP and their relationship with patient mortality..

**Methods:**

In our previous work, we introduced a novel measure of brain–heart interaction termed brain–heart crosstalks (*ct*_*np*_), as well as two additional brain–heart crosstalks indicators [mutual information ($mi_{ct}$) and average edge overlap (*ω*_*ct*_)] obtained through a complex network modeling of the brain–heart system. These measures are based on identification of simultaneous increase of HR and ICP. In this article, we investigated the relationship of these novel indicators with respect to mortality in a multicenter TBI cohort, as part of the Collaborative European Neurotrauma Effectiveness Research in TBI high-resolution work package.

**Results:**

A total of 226 patients with TBI were included in this cohort. The data set included monitored parameters (ICP and HR), as well as laboratory, demographics, and clinical information. The number of detected brain–heart crosstalks varied (mean 58, standard deviation 57). The Kruskal–Wallis test comparing brain–heart crosstalks measures of survivors and nonsurvivors showed statistically significant differences between the two distributions (*p* values: 0.02 for $mi_{ct}$, 0.005 for *ct*_*np*_ and 0.006 for *ω*_*ct*_). An inverse correlation was found, computed using the point biserial correlation technique, between the three new measures and mortality: − 0.13 for *ct*_*np*_ (*p* value 0.04), − 0.19 for *ω*_*ct*_ (*p* value 0.002969) and − 0.09 for $mi_{ct}$ (*p* value 0.1396). The measures were then introduced into the logistic regression framework, along with a set of input predictors made of clinical, demographic, computed tomography (CT), and lab variables. The prediction models were obtained by dividing the original cohort into four age groups (16–29, 30–49, 50–65, and 65–85 years of age) to properly treat with the age confounding factor. The best performing models were for age groups 16–29, 50–65, and 65–85, with the deviance of ratio explaining more than 80% in all the three cases. The presence of an inverse relationship between brain–heart crosstalks and mortality was also confirmed.

**Conclusions:**

The presence of a negative relationship between mortality and brain–heart crosstalks indicators suggests that a healthy brain–cardiovascular interaction plays a role in TBI.

## Introduction

Severe traumatic brain injury (TBI) is a leading cause of death and disability worldwide and across all ages [1], and the mortality rate seems to be unchanged over the past 25 years [2]. TBI affects 50 to 60 millions of new cases per year, with 2.5 million occurring in Europe, and this is the reason why it has been declared a priority for public health policy [3]. Efforts should be put into investments in research and other disciplines, such as clinical management and prevention policies. The clinical challenge is represented by the fact that the patient group is extremely heterogeneous and the pathology is highly dynamic [4]. Therefore, treatment protocols and prediction models are difficult to assess.

The critical care management in the acute phase is focused on reducing the probability and impact of secondary insults, which develop over time as a consequence of raised intracranial pressure (ICP) and/or reduced cerebral perfusion pressure, among other mechanisms [4]. Therefore, continuous ICP monitoring is recommended by international guidelines as a standard of care in all surviving patients with severe TBI to provide information for ICP-directed therapy [5, 6].

Along with ICP, a great deal of neuromonitoring techniques and imaging modalities can be used to improve the understanding of intracranial pathophysiology, which, as mentioned before, is highly heterogeneous and dynamic [4]. Ultimately, more precise targets for therapies could be suggested with this integrated approach. In this perspective, the interaction between the brain and other organs has been suggested as one of the mechanisms that could potentially explain the complexity of this pathology.

In particular, much attention has been given in recent years to the study of interactions existing between the brain and heart [7–9]. The dynamical interplay between the two organs is thought to ensure physiological functions and to be involved in pathological conditions, too [7]. For example, in the work by Valenza et al. [7], the authors describe episodes of paroxysmal sympathetic hyperactivity, which often happen when there is a severe axonal injury. In the work by Valenza et al. [7], the authors describe paroxysmal sympathetic hyperactivity in the postresuscitation syndromes after serious anoxic-ischemic brain insults. An interesting pattern of frequency of these paroxysms has been noticed, as described in detail in the work by Valenza et al. [7]. These observations show the importance that the brain–heart coupling has in pathological events. As a consequence, efforts have been made toward exploring analytical methodologies that could tackle this phenomenon, with the final goal of developing metrics describing the brain–heart interaction [8, 10].

Nevertheless, this area is still wide open for investigation, particularly in TBI. In the work by Gao et al. [9], the authors presented an interesting analysis of interaction between brain and heart measures, showing the presence of interaction and Granger causality between ICP, mean arterial pressure and heart rate (HR). In our previous studies, we derived an HR–ICP measure that we denominated “brain–heart crosstalks,” defined as transient elevations of ICP and HR that occur simultaneously [11]. In that work, we presented multiple novelties. For the first time, to the best of our knowledge, the measure of brain–heart crosstalks was defined. Moreover, we presented a novel sliding window method to detect the presence of these events. We studied the crosstalks defined as so in a pediatric population and subsequently in a single-center study with an adult cohort [12], where we also conducted a pilot analysis of the relationship of our novel interaction metric with mortality.

In a further work, we modeled the coupled HR–ICP system as a multilayer network [13] that can be imagined as a framework in which different channels of the same overall modeled structure are included [14]. In this framework, each channel is represented by a layer and each node can maintain different neighbors and characteristics across different domains. In recent years, multilayer networks have been fruitfully applied to a variety of fields such as, for example politics, medicine, economics, social interaction, and time series [14]. Its strength is the capability of modeling relationships across variables (i.e., layers), and therefore their interaction and integration. In our past work [13], to the best of our knowledge, we used for the first time a multilayer network approach to model the relationship between ICP and HR during brain–heart crosstalks events.

In this work, we aimed to integrate several brain–heart crosstalks measures and examine their role in the context of mortality prediction models, also taking into account clinical, demographics, and monitored features that collectively reflect the severity of primary injury. For this purpose, we used the Collaborative European Neurotrauma Effectiveness Research in TBI (CENTER-TBI) high-resolution intensive care unit (ICU) cohort of patients with TBI [15, 16]. Our main hypothesis was that the integrated brain–heart crosstalk measures are statistically related to outcome in patients with TBI.

## Methods

### Material

The material used in the present work included data collected in 226 patients with TBI as part of the high-resolution CENTER-TBI cohort [15, 16] . This subset consists of patients treated with no external ventricular drain. In the cohort analyzed, 50 patients died. The patient outcome was recorded at 6 months after injury. In Table 1 we present the variables we used as input features. Patients were recruited prospectively between the beginning of 2015 and the end of 2017 from 21 centers across Europe. All patients were admitted to the ICU for their TBI during the course of the study, with high-frequency digital signals recorded from their ICU monitors during the course of their ICU stay.

**Table 1:** Low resolution variables used as input features

Variable type	Description
Age	Age at the time of hospitalization
Sex	Sex information
Theater	Time between the traumatic event and when the monitoring device for ICP is inserted
Pupil	Pupil Reactivity Score
CT variables	CT_DAI versus SOL (presence of DAI vs. SOL), CT midline shift, CT_Sah (presence of Sah), CT_contusion, CT_depression of skull fractured, CT_basal cisterns absent compressed, CT_extradurahematoma
Lab variables	Data collected through a blood sample, at the time of hospitalization. The data available were the following: sodium, potassium, glucose, hemoglobin, white blood cell counts, lymphocytes, neutrophils, platelet, CRP, and albumin
Emergency department data	Emergency data information were available for adults at the time of arrival in hospital. These included arrival PH, lactate, arrival arterial CO_2 (mm Hg)

In the table the demographic, imaging, and admission variables retrieved as per version 2.0 of the CENTER-TBI database are described

CO_2_, carbon dioxide; CRP, C reactive protein; CT, computed tomography; DAI, diffuse axonal injury; ICP, intracranial pressure; PH, hydrogen ion concentration or acidity; Sah, subarachnoid hemorrhage; SOL, shift of the midline structures

All the patients had invasive ICP monitoring according to the Brain Trauma Foundation guidelines [6].

ICP was acquired using an intraparenchymal strain gauge probe (Codman ICP MicroSensor; Codman & Shurtleff Inc., Raynham, MA) or parenchymal fiber optic pressure sensor (Camino ICP Monitor, Integra Life Sciences, Plainsboro, NJ, US). Arterial blood pressure was obtained through either radial or femoral arterial lines connected to pressure transducers (Baxter Healthcare Corp. CardioVascular Group, Irvine, CA).

All signals were recorded using digital data transfer or digitized via an A/D converter (DT9801; Data Translation, Marlboro, MA), where appropriate, sampled at frequency of 100 Hz or higher, using the ICM + software (Cambridge Enterprise Ltd., Cambridge, UK) or Moberg CNS Monitor (Moberg Research Inc., Ambler, PA, USA) or a combination of both [17]. Signal artifacts were removed using both manual and with automated methods. Post-acquisition processing was conducted using ICM + (https://icmplus.neurosurg.cam.ac.uk). HR was determined by calculating the Fourier transform and finding the fundamental frequency of the arterial blood pressure waveforms over a 10-s window, updated every 10 s. 10-s moving average filter was applied. For each patient, we considered the whole monitored period available that corresponds to the whole duration of ICP monitoring, with initiation of recording within 24 h of the injury.

Low resolution data, such as demographic characteristics, admission, and injury related variables, were retrieved as per version 2.0 of the CENTER-TBI database. In Table 2, we present the summary of the variables describing the dataset used in the present work comparing distributions between the survivors and nonsurvivors.

**Table 2:** Overview of the variables used in the model

Features	Survivors (*n* = 176)	Nonsurvivors (*n* = 50)	*p* value
Age (mean ± SD)	42.98 ± 17.13	59.44 ± 16.9	< 0.05
Sex	34 females,142 males	12 females, 38 males	< 0.05
Pupil IMPACT score			
0	117	31	< 0.05
1	21	3	< 0.05
2	38	26	< 0.05
Arrival PH (mean ± SD)	4.62 ± 3.55	4.69 ± 3.55	> 0.05
Arrival lactate (mean ± SD)	2.50 ± 6.2	2.9 ± 6.8	> 0.05
Arrival art pCO2 mm hg (mean ± SD)	25.7 ± 21	26.05 ± 21.6	> 0.05
Sodium molL_1 (mean ± SD)	121.30 ± 47	120.308 ± 49	> 0.05
Potassium (mean ± SD)	3.21 ± 1.49	3.28 ± 1.58	> 0.05
Glucose (mean ± SD)	6.49 ± 4.5	6.62 ± 4.54	> 0.05
Hemoglobin dL (mean ± SD)	11.67 ± 4.26	11.02 ± 4.70	> 0.05
White blood cell pct_1 (mean ± SD)	11.38 ± 7.4	11.9 ± 8.4	> 0.05
Lymphocytes (mean ± SD)	7.45 ± 10.70	7.32 ± 9.85	> 0.05
Neutrophils (mean ± SD)	43.48 ± 39	49.47 ± 30	> 0.05
Platelet (mean ± SD)	181 ± 93	175.68 ± 93	> 0.05
CRP (mean ± SD)	3.49 ± 17	3.046 ± 7.87	> 0.05
Albumin (mean ± SD)	12 ± 17	15.93 ± 8.64	> 0.05
Theater (mean ± SD)	1.001 ± 0.69	0.98 ± 0.69	> 0.05
$ct_{np}$(mean ± SD)	0.17 ± 0.49	0.11 ± 0.16	< 0.05
$mi_{ct}$(mean ± SD)	0.55 ± 0.066	0.54 ± 0.08	> 0.05
$\omega_{ct}$(mean ± SD)	0.70 ± 0.09	0.65 ± 0.11	< 0.05
DAI			> 0.05
0	142	44	
1	34	6	
Midline shift			> 0.05
0	110	25	
1	66	25	
Subarachnoid hemorrhage			> 0.05
0	86	20	
1	10	6	
2	63	13	
Contusion			> 0.05
0	76	17	
1	86	23	
2	14	10	

We present here an overview of the variables used in the model, with distribution divided between survivors and nonsurvivors, and the associated *p* value of the two-sampled Student’s *t*-test between the vectors of the features evaluated in the two populations

CRP, C reactive protein; $ct_{np}$, brain–heart crosstalks; DAI, diffuse axonal injury; IMPACT, international mission for prognosis and analysis of clinical trials [23]; $mi_{ct}$, mutual information; pCO_2_, partial pressure of carbon dioxide; PH, hydrogen ion concentration or acidity; SD, standard deviation; *ω*_*ct*_, average edge overlap

### Methods

#### Brain–Heart Crosstalks Detection and Analysis

In our own previous work [11], we proposed an algorithm to detect the presence of brain–heart crosstalks given two time series of ICP and HR monitored in patients with TBI.

Briefly, the algorithm is based upon a sliding window approach. A window of 10 min of observations is considered jointly in HR and ICP. If a simultaneous increase in HR and ICP, of at least 20% with respect to the minimum value of the window frame, followed by a subsequent decrease in both, is detected, then a brain–heart crosstalk is counted. Such measure was defined to facilitate the study of patterns of interaction between HR and ICP and used as a proxy of brain and heart interaction. Full details of the algorithm can be found in [11]. In the present study we applied our own algorithm just described, to detect the total number of brain–heart crosstalks for adult patients with TBI.

**Fig. 1:**
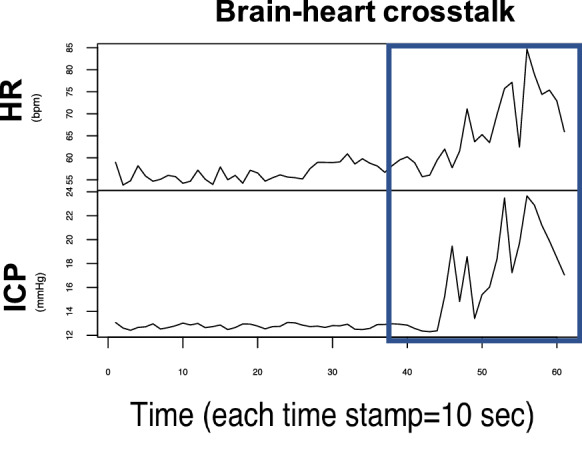
Example of one brain–heart crosstalk. In the figure we present time trends for more than 10 min of observations for HR and ICP. The blue rectangle denotes the presence of a simultaneous increase of HR and ICP. The event was detected using the sliding window approach. (*HR* heart rate, *ICP* intracranial pressure)

Since the length of observations varied for each patient (depending on various factors, for instance the duration of hospitalization), the raw number of detected brain–heart crosstalks was normalized by the total length of the analyzed time series (*ct*_*np*_). Figure 1 shows an example of brain–heart crosstalk, with the event marked by the blue rectangle.

In our later work [13], the behavior of ICP and HR time series, during brain–heart crosstalks events, was further investigated using multilayer network modeling. In particular, the system ICP-HR was modeled as a multiplex network, where each layer represents one of the time series. The conversion between time series and graphs works as follows: nodes represent time points, and an edge between two nodes is present when the criterion of the “horizontal visibility” is met [18]. Briefly, two nodes are connected if they “can see” each other horizontally, i.e., if all the values separating the two in time are of lower, or equal, magnitude. Because in our case we have the same sets of nodes for each layer, we used a special type of multilayer network named multiplex, where each node in a layer is connected to the corresponding one in the other layers.

**Fig. 2:**
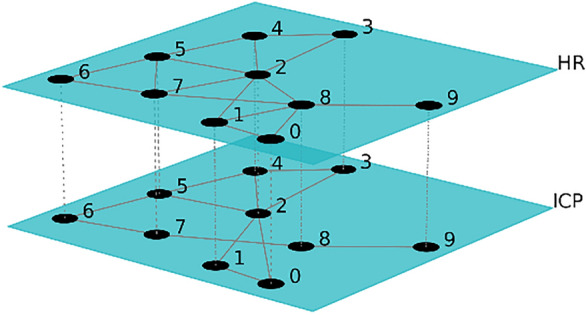
Figure showing the multilayer network model. Each layer is a time series. The top layer here represents HR, whereas the lowest layer represents ICP. Each node is a time stamp, and connections between two nodes are defined according to the horizontal visibility criterion [13]. (*HR* heart rate, *ICP* intracranial pressure)

An example of a multiplex network model, in which one layer represents ICP and the other represents HR, is shown in Fig. 2.

Using the complex network approach, we obtained two network measures: the interlayer mutual information ($${mi}_{ct})$$ and the average edge overlap $${(\omega }_{ct}$$). The average edge overlap $${\omega }_{ct}$$ quantifies the coherence of the overall graph, and the higher it is, the higher the coherence of the graph layers. The interlayer mutual information $${mi}_{ct}$$ models the degree distribution of the two graphs. Because one measure reflects the edges, and the other focuses on the nodes, the two measures complement each other, and they report a complete picture of the joint behavior of the two analyzed time series. They were therefore chosen as representatives of the graphs corresponding to the detected brain–heart crosstalk event windows. Complete details on the whole multiplex modeling can be found in our previous work [13].

The three defined measures of crosstalks, i.e., the count ${ct}_{np}$, the interlayer mutual information ${mi}_{ct}$ and the edge overlap ${\omega }_{ct}$, were added to the set of predictive features in the mortality model.

#### Mortality Model Predictions

In order to build a predictive mortality model we applied an elastic-net logistic regression machine learning approach [19, 20] obtaining a model for each of the four age groups in which the population was segmented, as described in the Results section. The machine learning approach used, is a slight modification of the standard logistic regression, enriched with a lasso and a ridge regression to overcome multicollinearity issues [19, 20]. It also performs an automatic features selection, embedded in the optimization resolution problem. Two parameters need to be set for the elastic-net model: $\lambda$ and $\alpha$, which are respectively used to tune the lasso and ridge contribution in the optimization function. In our experiment $\alpha$=0.5 and $\lambda$ was defined through a leave one out cross validation procedure, in which turn n-1 number of observations are used as training set and the remaining one is used as test set. The $\lambda$ values selected for each model as well as the performances of each model (in terms of percentage of null deviance explained) are specified in Table 3, at the top of each specific section discussing the model for each age range. Each model was fed with the set of input features described in Table 1 together with the newly introduced brain–heart crosstalk indicators.

**Table 3:** $\lambda$, percentages null deviance and coefficients selected by the elastic-net model are shown in the figure

Variables	Age 16–29 model, $\lambda$ = 0.019, dev. ratio: 0.74	Age 30–49 model, $\lambda$ = 0.04, dev. ratio: 0.47	Age 50–64 model, $\lambda$ = 0.0002, dev. ratio: 0.92	Age 65–85, model, $\lambda$ = 0.00003, dev. ratio: 0.99
	Selected coefficients	Selected coefficients	Selected coefficients	Selected coefficients
Intercept	− 4.27	− 2.46	− 8.3	− 0.81
Age			− 2.56	6.31
Sex		0.001	3.27	− 0.82
Pupil	0.006	0.23	0.60	4.20
Arrival PH	0.11	0.32	8.49	− 5.71
Lactate	0.2		− 8.09	7.9
Arrival arterial CO_2	0.18	0.33	− 8.29	− 1.1
Sodium		0.01	− 0.56	− 4.9
Potassium			− 2.69	2.71
Glucose	− 0.009		3.1	− 4.59
Hemoglobin	− 0.44		0.40	− 3.12
White blood cell counts			1.33	− 3.68
Lymphocytes			− 1.26	10.92
Neutrophils			− 2.03	− 1.71
Platelet	− 0.10		2.55	3.22
CRP	0.34	0.099	− 2.92	0.37
Albumin	1.01		5.32	2.60
CT_DAI versus SOL			− 7.90	3.99
CT_MidlineShift			− 2.31	− 4.41
CT_Sah	1.04		− 3.38	− 2.79
CT_Contusion		0.12	6.51	− 3.25
CT_DeprSkullFract		0.52	− 3.94	2.87
CT_BasalCisternsAbsentCompressed			− 3.41	0.95
CT_ExtraduralHema		− 0.23	− 3.79	3.65
Theater		0.38	− 2.13	− 1.41
$ct_{np}$	− 0.08		− 3.39	− 3.46
$mi_{ct}$	− 1.10		3.32	4.95
$\omega_{ct}$	− 0.11	− 0.37	− 0.78	− 1.84

At the top of the table, we can see the age ranges they refer to. Coefficients are specified in the second column of each table. Dots (.) in the coefficients column mean that the variable was not selected as significant

CRP, C reactive protein; CT, computed tomography; $ct_{np}$, brain–heart crosstalks; $mi_{ct}$, mutual information; PH, hydrogen ion concentration or acidity; Sah, subarachnoid hemorrhage; SOL, shift of the midline structures; *ω*_*ct*_, average edge overlap

### Statistical Analysis

The brain–heart crosstalks algorithm as well as the statistical analysis was conducted using R software [21] version 3.6.0. The point biserial correlation test was performed to verify the correlation between brain–heart crosstalks measures and mortality. Pearson correlation coefficient was used to test correlation between input features. Kruskal–Wallis test was used to verify the presence of statistically significant difference between distributions of brain–heart crosstalks variables in surviving and nonsurviving patients with TBI.

## Results

### Crosstalks and Preliminary Statistical Analysis

**Fig. 3:**
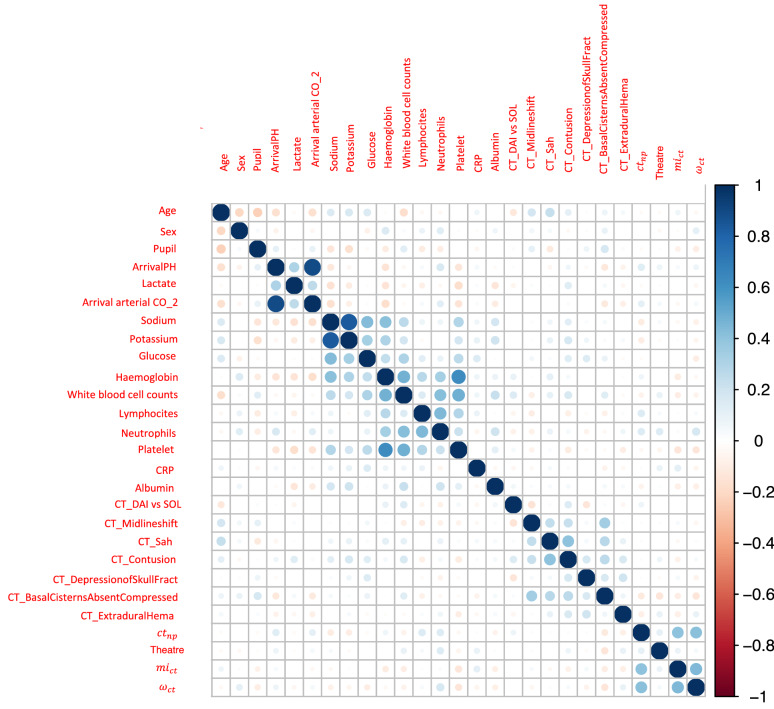
Pearson correlation of the predictive variables for the adult cohort. The matrix is symmetric, and we included here all the clinical, lab, imaging, as well as brain–heart crosstalks variables. (*CRP* C reactive protein; *CT* computed tomography; $ct_{np}$ brain–heart crosstalks; *DAI* diffuse axonal injury; $mi_{ct}$ mutual information; *SOL* shift of the midline structures; *ω*_*ct*_ average edge overlap)

A mean number of 57 brain–heart crosstalks, per patient, with a high standard deviation of 58, were detected. The value of brain–heart crosstalks was normalized by the length of the time series observed per patient, and its correlations with the other predictive variables are reported in Fig. 3. The apparent “squared patterns” of correlations, starting from the left upper corner of the correlation matrix, are likely to correspond to similar features, for instance lab blood samples analysis or CT features. These subgroups appear to be naturally correlated, although other correlations, even if milder, appear in the rest of the plot.

**Fig. 4:**
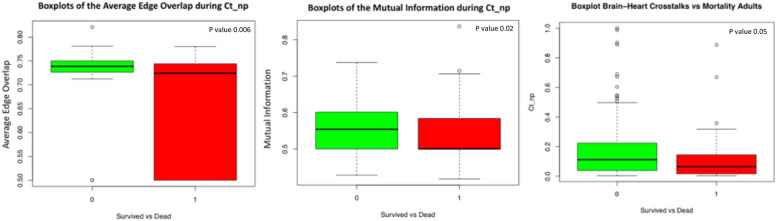
Distribution of the brain–heart crosstalks measures for survivors and nonsurvivors. The *p* value of the Kruskal–Wallis test performed between the two distributions is also shown in the figure. ( $ct_{np}$ brain–heart crosstalks)

The relationship between mortality and brain–heart crosstalks was first examined using the distribution analysis of brain–heart crosstalks and related network measures, comparing survivors and nonsurvivors. Figure 4 shows the boxplots of the distributions of *ct*_*np*_, *ω*_*ct*_ and $mi_{ct}$ for the entire cohort (*n* = 226).

The point biserial correlation between mortality and *ct*_*np*_ in adults is − 0.13 with a significant *p* value of 0.04. This seems to suggest an inverse relationship existing between mortality and brain–heart crosstalks in the cohort analyzed. Same can be said for *ω*_*ct*_ which present − 0.19 as a point biserial correlation (with significant *p* value of 0.002) and for $mi_{ct}$ with − 0.09 (with a *p* value slightly more than the significant threshold, and equal to 0.13).

Moreover, as it can be appreciated from the *ct*_*np*_ boxplot in Fig. 4, the visual difference between the survivors and nonsurvivors distribution of the brain–heart crosstalks measures was confirmed statistically. The Kruskal–Wallis test to compare the distributions of the three measures returned significant *p* values for all of them: 0.02 for ${mi}_{ct}$, 0.005 for *ct*_*np*_ and 0.006 for *ω*_*ct*_.

### Mortality Model

**Fig. 5:**
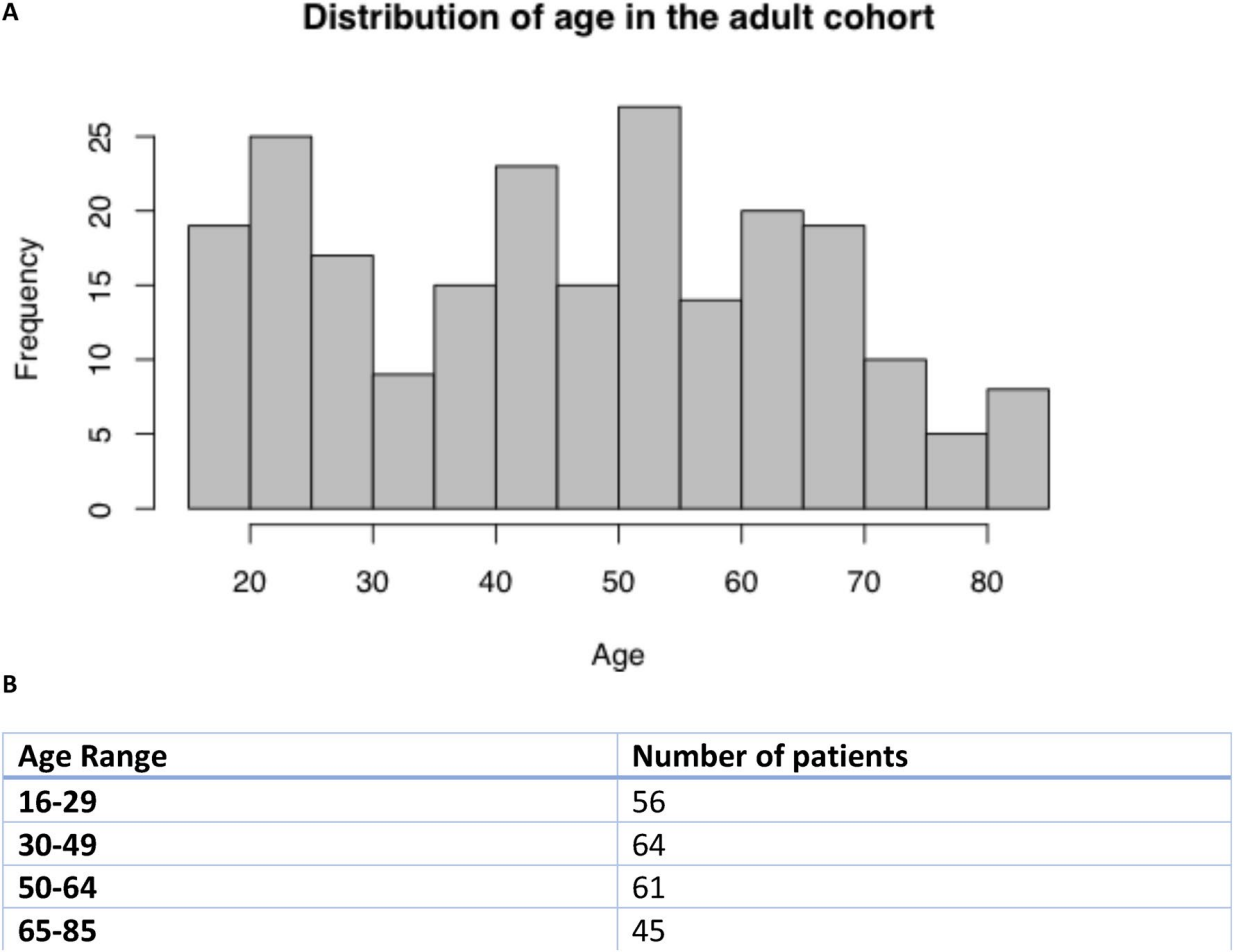
Distribution of age in the adult cohort. **A** Histogram of age distribution in the high-resolution CENTER-TBI cohort on top. **B** Age ranges groups and their relative numerosity, in the lower part of the figure

The age distribution of the patients included in this study ranged between 16 and 85 years old and is presented in Fig. 5 panel a. Age has been shown to have a significant impact toward TBI prognostic and outcome prediction [22], also because of its influence on the likelihood of comorbidities. To decrease the heterogeneity of patients analyzed in the mortality models, the whole dataset was divided into four age category subgroups. In panel b of Fig. 5 we show the number of patients with the associated age range after the splitting process, whose composition is homogeneous within groups and heterogeneous across groups. In other words, the division into four age groups was performed in a way in which we could obtain four groups of homogeneously aged patients, with comparable sample size, in order to be statistically possible the comparison between groups. In this way we could still include age as a predictor variable in each of the four models, while avoiding using it as a confounding factor, when considering the entire population with no age distinction. In Table 3, we show the results for the models fitted for each age group, including their deviance ratio explained, in the header of each table. Coefficients for all the features in the model are presented. The best fitting models appears to be for the age populations range aged 16–29, 50–64, and 65–85. Indeed, the deviance ratio is high which means that the predictors included in the model explain with very high reliability the mortality outcome. Interestingly, the negative relationship between mortality and *ct*_*np*_ in all the age populations is confirmed as well as the one between mortality and the average edge overlap during crosstalks. In so far as the other populations are concerned, the best fit of the model appears to be for the age range 65–85. We also performed experiments with and without the newly introduced brain–heart crosstalks measures. The deviance ratio actually does improve for the case of age range 16–29 (from 49 to 79%) and 65–85 (from 93 to 99%). In Table 3, we only reported the value of the significant coefficients. If a dot is in the corresponding cell, it means the coefficient is not significant. We also performed further experiments using only the IMPACT (International Mission for Prognosis and Analysis of Clinical Trials [23]) variable scores as predictors and adding subsequently also brain–heart crosstalks measures to the model to evaluate if the IMPACT variables would have benefited from the introduction of our brain–heart crosstalks variables. In particular, we used the standard IMPACT variables of age, motor score, pupils, hypoxia, hypotension, CT variables, glucose, and hemoglobin for predicting mortality using the logistic regression approach and subsequently we added the brain–heart crosstalks measures to the IMPACT scores and evaluated performances of the models so obtained in predicting mortality of the patients. In all the cases the deviance ratio of the model improved, except for the group 50–64 where it remained the same.

**Fig. 6:**
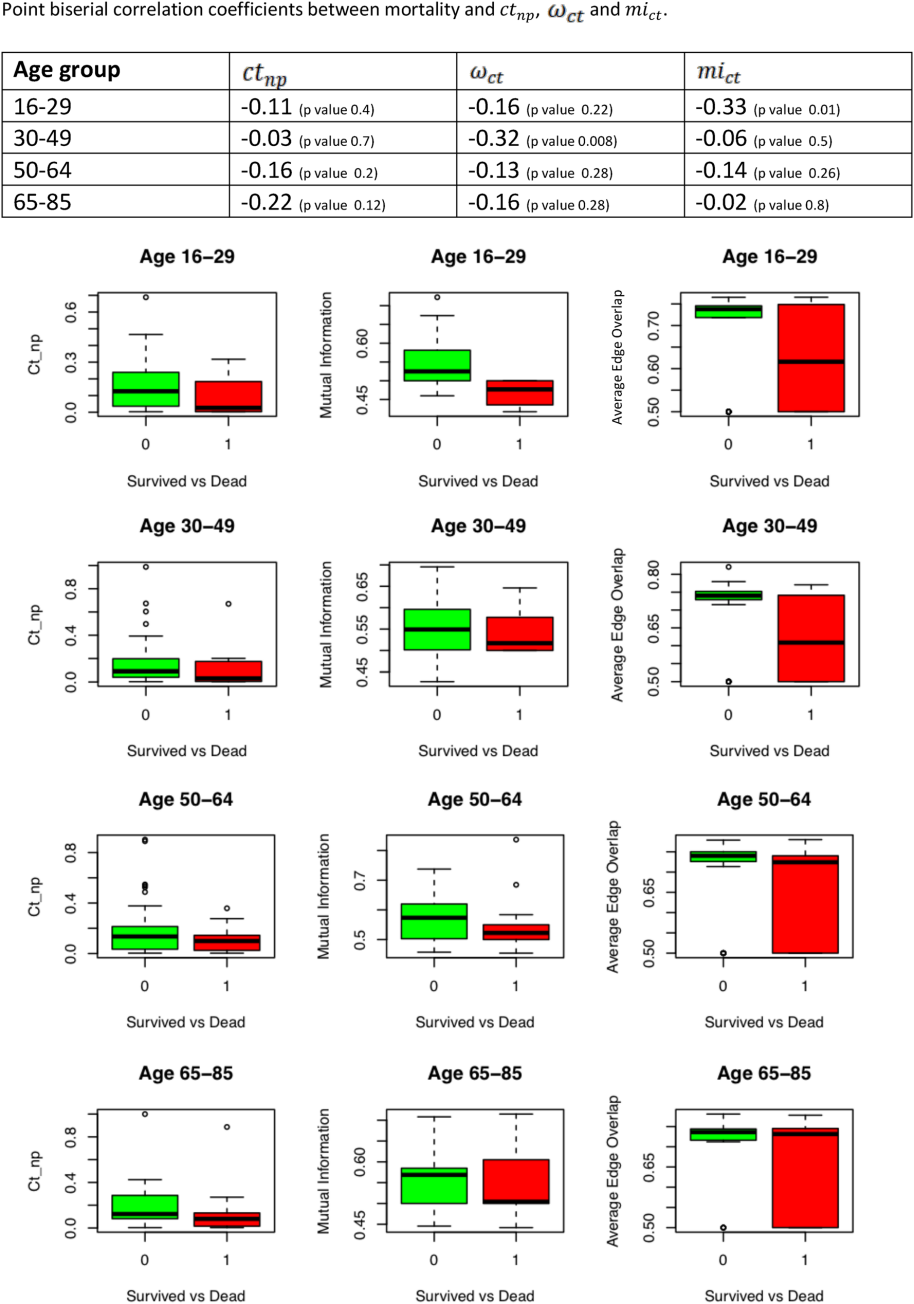
Table with the point biserial correlations tests between brain–heart crosstalks measures and mortality for the four age groups (top). In the bottom of the figure, we present the boxplots for the $ct_{np}$, $\omega_{ct}$ and $mi_{{ct{ }}}$ for the survivors and nonsurvivors in the four age groups defined. ($ct_{np}$, brain–heart crosstalks; $mi_{ct}$, mutual information; *ω*_*ct*_, average edge overlap)

To investigate further the correlation between brain–heart crosstalks and mortality, the point biserial correlation coefficients between mortality and the brain–heart crosstalks measures were also obtained. The results, summarized in Fig. 6, shows the point biserial correlation coefficients between the network measures and mortality, exhibiting negative sign in almost all of them. Moreover, in Fig. 6 we present the boxplots of the three newly introduced brain–heart crosstalks measures for survivors and nonsurvivors in the four age groups. In Table 4 we report the *p* values of the Kruskal–Wallis test performed for the brain–heart crosstalks measures of the four age groups, comparing survivors and nonsurvivors.

**Table 4:** Table showing the Kruskal–Wallis tests between survivors and nonsurvivors of each age group analyzed

Age group	$ct_{np}$(KW *p* value)	*ω*_*ct*_ (KW *p* value)	$mi_{ct}$(KW *p* value)
16–29	0.21	0.52	0.01*
30–49	0.17	0.05*	0.49
50–64	0.3	0.09	0.08
65–85	0.03*	0.42	0.62

*Significant *p* values

*KW* Kruskal–Wallis

## Discussion

In this article, we investigated the relationship between mortality and events of interaction between brain and heart in patients with TBI, named brain–heart crosstalks.

Brain–heart crosstalks were introduced in our previous work [11]. This novel metric detects events of simultaneous increases of ICP and HR.

Brain–heart crosstalks measures were further extended, through a complex network modeling [13], which led to the computation of two measures for the ICP-HR system: average edge overlap ($\omega_{ct} )$ and mutual information ($mi_{ct}$), which give indication of the behavior of the system during brain–heart crosstalks, as described more in details in the methods section.

From the current study, we could see that the results suggested an inverse relationship between brain–heart crosstalks measures and mortality. The point biserial correlation coefficient was always negative, for the three network measures and mortality. This was true for the case in which we computed it using the 226 patients and when segmenting the population in four age groups. In fact, for our mortality model we divided the population in age groups in the attempt of decreasing the heterogeneity brought by this clinical variable. Important differences in terms of comorbidities, type of lesions and outcome prospective are related to age, particularly in the face of the TBI population aging in western countries.

The first indication of a negative relationship between mortality and brain–heart crosstalk measure, was further confirmed by the Kruskal–Wallis tests between survivors and nonsurvivors distribution of those measures in the whole dataset. In addition, when segmenting the original population, at least one of the brain–heart crosstalks measures was statistically significantly different between survivors and nonsurvivors for each age group, except for the age group 50–64. However, for this age group in the logistic regression model, the coefficient associated to average edge overlap, and the normalized numbers of crosstalks ${ct}_{np}$, was statistically significant and with a negative sign. Logistic elastic-net prediction models appeared to have a good fitting to the data, as the percentage of deviance ratio explained is sufficiently high. This was true except for the age group 30–49. These age-related findings are difficult to explain based on the dataset studied and warrant further investigation in a larger group.

Another important aspect to be considered is that the sign of the ${ct}_{np}$ coefficient was consistently negative for all the four age groups. Given these results, we can confirm that the higher the number of brain–heart crosstalks, the lower is the probability of mortality. This result is confirmed independently of the age group to which the individuals belong to.

Similar behavior was exhibited by the average edge overlap, while for the mutual information two age groups showed positive relationship between mortality and the indicator. This evidence will need further investigation in the future.

Overall, our results suggest that the newly introduced variables of brain–heart crosstalks, might be considered as biomarkers of a healthy brain–heart interaction and therefore the assessment of these biomarkers could play a role in understanding the pathological mechanisms in TBI. The complexity of such a disease is indeed difficult to explain with simply measuring mean ICP values and multimodality physiological monitoring techniques have been developed to improve our understanding and are implemented in the clinical practice. In this perspective, considering brain–heart crosstalks along with other physiology measures in integrated protocols may be a promising approach.

For what concerns the other variables of the prediction models fitted, age came as a statistically significant element only for the two older age groups, but not for the two younger ones. This is in line with findings suggested by the literature, where there is evidence that older age might be a factor worsening the outcome of TBI, because of the comorbidities associated along with other factors [22]. Moreover, from the fitted models, as we can see in Table 3 for the older populations, almost all the variables have statistically significant coefficients, unlike younger population groups. This is consistent with the fact that the model for the two older population groups showed a better fitting than the younger population groups. Possibly, the higher prevalence of cardiologic preexisting disease may explain the relevance of the brain–heart interplay in the elderly.

As a final consideration our analysis shed light on the possibility of integrating brain–heart crosstalks measures into the existing TBI prognostic models. A very well-known prognostic model is the IMPACT score [23], where admission features as for instance age, motor score, pupils, hypoxia, hypotension, CT variables, glucose, and hemoglobin are used to evaluate patient’s condition at the time of hospitalization. Some work has been done for what concerns more complex prognostic models development, but to the best of our knowledge, none has ever taken into account variables expressing the interaction between brain and heart [24]. Therefore, our analysis opens the possibility for implementing the TBI prognostic models with brain–heart interaction measures, and hopefully increasing their precision.

## Conclusions

We found an inverse relationship between the number of brain–heart crosstalks events and mortality in patients with TBI. We also applied two further indicators of brain–heart crosstalks from a complex network modeling of the ICP-HR system, which showed similar inverse behavior with mortality. The inverse relationship was also confirmed when fitting the input features into a mortality prediction model.

We are currently investigating how brain–heart crosstalks relate to other physiological variables (e.g., autonomic nervous activities) or clinical variables (treatments a patient receives, airways suctioning, presence of intracranial hypertension or impaired autoregulation). In so far as limitations are concerned, we believe that the division into four age groups was necessary to avoid confounding effects due to patients’ wide age differences. However, we are aware that this reduces the number of observations in each group when fitting the related models thus potentially resulting in fitting bias. Therefore, we plan to extend the study to a larger cohort of patients to explore further the model proposed. Currently we have started a preliminary analysis concerning the evidence of a causality existing between HR and ICP during brain–heart crosstalks, which could lead to another important contribution toward the use of brain–heart crosstalks in clinical practice. For future work, it would also be interesting understanding the possible relationship between different events (such as only raised ICP) with mortality.

Furthermore, we would like to extend the use of the crosstalks as a real-time digital biomarker in patients with TBI, through the use of an ICM+-Python plugin interface [17] running at the bedside. This would ultimately facilitate the use our new metric in the individualized management of this patients. We are in the process of implementing in Python the code developed initially in R.

## References

[1] Maas AIR, Menon DK, Adelson PD, Andelic N, Bell MJ, Belli A (2017). Traumatic brain injury: integrated approaches to improve prevention, clinical care, and research. Lancet Neurol.

[2] Donnelly J, Czosnyka M, Adams H, Cardim D, Kolias AG, Zeiler FA (2019). Twenty-five years of intracranial pressure monitoring after severe traumatic brain injury: a retrospective, single-center analysis. Neurosurgery.

[3] Quaglio G, Gallucci M, Brand H, Dawood A, Cobello F (2017). Traumatic brain injury: a priority for public health policy. Lancet Neurol.

[4] Stocchetti N, Carbonara M, Citerio G, Ercole A, Skrifvars MB, Smielewski P (2017). Severe traumatic brain injury: targeted management in the intensive care unit. Lancet Neurol.

[5] Hawryluk GWJ, Aguilera S, Buki A, Bulger E, Citerio G, Cooper DJ, et al. A management algorithm for patients with intracranial pressure monitoring: the Seattle international severe traumatic brain injury consensus conference (SIBICC). In: Intensive care medicine [Internet]. Springer; 2019 [cited 2020 Oct 21]. p. 1783–94. 10.1007/s00134-019-05805-9. 10.1007/s00134-019-05805-9 PMC6863785 31659383

[6] Carney N, Totten AM, Hawryluk GWJ, Bell MJ, Bratton SL, Chesnut R, et al. Guidelines for the management of severe traumatic brain injury 4th edition. 2016 [cited 2017 Dec 21]. https://braintrauma.org/uploads/03/12/Guidelines_for_Management_of_Severe_TBI_4th_Edition.pdf.

[7] Valenza G, Toschi N, Barbieri R. Uncovering brain-heart information through advanced signal and image processing [Internet]. Vol. 374, Philosophical Transactions of the Royal Society A: Mathematical, Physical and Engineering Sciences. Royal Society of London; 2016 [cited 2020 Oct 29]. https://pubmed.ncbi.nlm.nih.gov/27044995/. 10.1098/rsta.2016.0020 PMC4822450 27044995

[8] Silvani A, Calandra-Buonaura G, Dampney RAL, Cortelli P. Brain-heart interactions: Physiology and clinical implications [Internet]. Vol. 374, Philosophical Transactions of the Royal Society A: Mathematical, Physical and Engineering Sciences. Royal Society of London; 2016 [cited 2020 Oct 29]. https://pubmed.ncbi.nlm.nih.gov/27044998/. 10.1098/rsta.2015.0181 27044998

[9] Gao L, Smielewski P, Czosnyka M, Ercole A (2017). Early asymmetric cardio-cerebral causality and outcome after severe traumatic brain injury. J Neurotrauma.

[10] Baguley IJ, Heriseanu RE, Cameron ID, Nott MT, Slewa-Younan S (2008). A critical review of the pathophysiology of dysautonomia following traumatic brain injury. Neurocrit Care.

[11] Dimitri GM, Agrawal S, Young A, Donnelly J, Liu X, Smielewski P, et al. Simultaneous transients of intracranial pressure and heart rate in traumatic brain injury: Methods of analysis. In: Acta Neurochirurgica, Supplementum [Internet]. Springer-Verlag Wien; 2018 [cited 2020 Oct 29]. p. 147–51. https://pubmed.ncbi.nlm.nih.gov/29492551/. 10.1007/978-3-319-65798-1_31 29492551

[12] Dimitri GM, Beqiri E, Czosnyka M, Ercole A, Smielewski P, Liò P. Analysing cardio-cerebral crosstalks in an adult cohort from CENTER-TBI. Acta neurochirurgica Supplement, in press. 2020;(1). 10.1007/978-3-030-59436-7_9 33839815

[13] Dimitri GM, Agrawal S, Young A, Donnelly J, Liu X, Smielewski P, et al. A multiplex network approach for the analysis of intracranial pressure and heart rate data in traumatic brain injured patients. Applied Network Science [Internet]. 2017 Dec 1 [cited 2020 Oct 29];2(1). Available from: https://pubmed.ncbi.nlm.nih.gov/30443583/. 10.1007/s41109-017-0050-3 PMC6214250 30443583

[14] Boccaletti S, Bianconi G, Criado R, del Genio CI, Gómez-Gardeñes J, Romance M (2014). The structure and dynamics of multilayer networks. Phys Rep.

[15] Maas AIR, Menon DK, Steyerberg EW, Citerio G, Lecky F, Manley GT (2015). Collaborative European NeuroTrauma effectiveness research in traumatic brain injury (CENTER-TBI). Neurosurgery.

[16] Steyerberg EW, Wiegers E, Sewalt C (2019). Case-mix, care pathways, and outcomes in patients with traumatic brain injury in Europe from the CENTER-TBI cohort: an epidemiological study. Lancet Neurol.

[17] Placek MM, Khellaf A, Thiemann B, Cabeleira M, Smielewski P (2020). Python-embedded plugin implementation in ICM+: novel tools for neuromonitoring time series analysis with examples using CENTER-TBI datasets. Acta Neurochirurgica Suppl.

[18] Luque B, Lacasa L, Ballesteros F, Luque J. Horizontal visibility graphs: Exact results for random time series. Phys Rev E Stat Nonlinear Soft Matter Phys [Internet]. 2009 Oct 7 [cited 2020 Oct 29];80(4). Available from: https://pubmed.ncbi.nlm.nih.gov/19905386/. 10.1103/PhysRevE.80.046103 19905386

[19] Tibshirani R. Regression shrinkage and selection via the Lasso [Internet]. JSTOR. [cited 2020 Oct 29]. Available from: https://www.jstor.org/stable/2346178?seq=1#metadata_info_tab_contents.

[20] Hoerl AE, Kennard RW. Ridge regression: biased estimation for. Vol. 12. 1970.

[21] R: The R Project for Statistical Computing [Internet]. [cited 2020 Oct 29]. https://www.r-project.org/.

[22] Stocchetti N, Paternò R, Citerio G, Beretta L, Colombo A (2012). Traumatic brain injury in an aging population. J Neurotrauma.

[23] Steyerberg EW, Mushkudiani N, Perel P, Butcher I, Lu J, McHugh GS (2008). Predicting outcome after traumatic brain injury: development and international validation of prognostic scores based on admission characteristics. PLoS Med.

[24] Mushkudiani NA, Hukkelhoven CWPM, Hernández AV, Murray GD, Choi SC, Maas AIR (2008). A systematic review finds methodological improvements necessary for prognostic models in determining traumatic brain injury outcomes. J Clin Epidemiol.

